# Trade‐Offs Between Forage Availability, Accessibility, and Predation Risk on Winter Foraging Strategies of Wood Bison (*Bison bison athabascae*)

**DOI:** 10.1002/ece3.70385

**Published:** 2024-10-08

**Authors:** Garrett J. Rawleigh, Mark A. Edwards, Darren Epperson, Scott E. Nielsen

**Affiliations:** ^1^ Department of Renewable Resources University of Alberta Edmonton Alberta Canada; ^2^ Office of the Chief Scientist, Environment and Protected Areas Government of Alberta Edmonton Alberta Canada

**Keywords:** energy maximization hypothesis, foraging ecology, optimal foraging theory, wood bison

## Abstract

Optimal foraging theory (OFT) and the energy maximization hypothesis (EMH) have long been essential when examining wildlife habitat selection. At high latitudes and altitudes, animals in winter face greater limitations in availability and accessibility of forage. Here we explore the foraging behavior of wood bison (*Bison bison athabascae*) during winter within the Ronald Lake bison herd in northeastern Alberta, Canada, and examine the trade‐offs they face due to limitations in forage abundance and availability (snow conditions), as well as the need to minimize predation risk. We used Global Positioning System (GPS) location data collected from 70 female wood bison to identify winter foraging sites and craters selected by bison to access forage beneath the snow. Within wetlands used by bison we selected 190 pairs of used (foraged) and random (available) sites to test eight a priori hypotheses explaining how bison traded‐off between forage availability, accessibility, and minimizing predation risk. We found with matched‐paired logistic regression that *Carex atherodes* was 1.21‐times more likely to be selected per unit increase in ground cover, compared to 1.17‐times per unit ground cover for *C. aquatilis* and *C. utriculata*. However, all *Carex* species showed an increase in selection when cover was > 50% cover within individual craters. While the importance of *Carex* was clear, forage site selection was still inversely related to snow depth. There is also a neutralizing combined effect of snow depth and *Carex* species ground cover which suggests that bison maximized their energy return by avoiding areas with deep snow (> 30 cm) that demanded intensive cratering, even when highly selected forage was accessible beneath. Avoidance of forage areas with deep snow demonstrates that wood bison employed a foraging strategy that considers both forage availability and environmental conditions, with snow depth being a limiting factor. We highlight the relationship between optimal foraging based on food availability and the trade‐offs within an energy restrictive winter season, furthering the understanding of how large herbivores forage strategically to maximize energy intake in northern environments.

## Graphical abstract

We studied the winter foraging ecology of wood bison in northeastern Alberta. Finding that wood bison select for sedge species, but, increased snow depths lead to decreased selection despite the presence of highly selected forage species beneath. This study highlights bison strategies to maximize energy in northern winters.

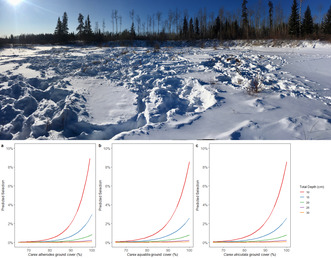

## 1 Introduction

The mechanisms behind wildlife foraging decisions have long been discussed by ecologists (MacArthur and Pianka 1966; Emlen 1968; Pyke 1984). Foraging results in gathering energy from foods, leading many to link evolutionary fitness to optimized foraging strategies. Optimal foraging theory (OFT) frames foraging behaviors as a function of peak individual fitness achieved by maximizing the individual rate of energy intake (MacArthur and Pianka 1966; Emlen 1968). However, a closer examination of foraging behavior has highlighted several constraints to OFT, including competition, predator avoidance, and nutrient acquisition (Pyke 1984; Hughes 1990). For instance, the energy maximization hypothesis (EMH) accounts for these constraints and considers how organisms maximize their total energy gain while minimizing energy loss (Pyke 1984; Hughes 1990). Constraints on foraging are specifically relevant during winter at high latitudes and altitudes, with reduced forage availability and harsh environmental conditions increasing stress on individuals.

Snow cover and dormancy of most primary producers during winters at high latitudes and altitudes reduces forage available to large ungulates, resulting in an adjustment of foraging strategies (Strong and Gates 2009; Jung 2015). Wood bison (*Bison bison athabascae*) are large grazing ungulates with populations scattered from northwestern Canada through Alaska and thus are exposed to winter conditions of forage‐limitation. OFT predicts that wood bison foraging decisions are based on the availability of foods that maximize energy (Emlen 1968; MacArthur and Pianka 1966; Fortin et al. 2003). Wood bison respond to seasonal forage availability by switching to a graminoid‐dominant diet during winter in frozen wet meadows (Larter and Gates 1991; Hecker, Edwards, and Nielsen 2021). Bison particularly select for wetlands dominated by *Carex atherodes*, a species of sedge with a high availability of digestible energy during winter (Courant and Fortin 2012a, 2012b). Thus, OFT predicts bison forage site selection will be positively related to increased abundance of *C. atherodes* and other sedge species. However, this prediction could be limited by constraints, such as snow conditions limiting access to forage, and predation minimizing behaviors influencing forage patch selection (Hernández and Laundré 2005; Bruggeman et al. 2008).

Deeper snow and harder snow crusts require greater energetic costs for movement and foraging, leading to energy maximization strategies (Bruggeman et al. 2008). For example, elk (*Cervus elaphus*) foraging is limited by snow depth, and muskoxen (*Ovibos moschatus*) increase their energy expenditure cratering for food in deep snow (Sweeney and Sweeney 1984; Schaefer and Messier 1995a). Likewise, wood bison select shallower snow while moving and foraging, decreasing movement rates at high snow depths and during cold winter temperatures (Bruggeman et al. 2008; Sheppard et al. 2021). Predation pressure can further alter foraging behaviors, as prey animals adopt behaviors that reduce predation risk, including increasing vigilance and relocating to less risky habitats (Nelson and Mech 1986; Harvey and Fortin 2013; Mpemba et al. 2019). Boreal ungulates, like woodland caribou (*Rangifer tarandus caribou*), have been shown to spend more time foraging in locations with high adjacent cover (Mason and Fortin 2017). Gray wolves (*Canis lupus*) are the primary predator of wood bison (Oosenbrug, Carbyn, and Herrero 1985; Carbyn and Trottier 1988; Smith et al. 2000; Dewart et al. 2020). For plains bison (*B. b. bison*), the presence of wolves has been shown to limit their time in areas of high‐quality forage, spending more time in smaller forage patches with access to cover (Harvey and Fortin 2013; Simon, Cherry, and Fortin 2019). Prey species also avoid areas preferred by predators (Doncaster 1994), and given wolves often use open spaces to pursue prey, wetland meadows pose a predation risk despite being high in forage availability (Bergman et al. 2006). Foraging in wetland meadows balances the risk of being chased towards forest edges and being near camouflaging cover and may provide an evolutionarily effective trade‐off for wood bison (Carbyn and Trottier 1988; Bergman et al. 2006).

Habitat selection and foraging behaviors have been studied in many ways with Johnson (1980) providing a hierarchical framework for studying the selection of resources. First order selection is at the geographic range level; the second order is at the selection of home ranges; the third order is at the selection of patches (feeding sites) within home ranges; and the fourth order is at the level of forage procurement within a feeding site (Johnson 1980). Foraging decisions within the feeding site can be understood by quantifying the competing factors that affect forage selection. For bison, these factors include food availability and environmental conditions like snow conditions or predation pressure. To determine how wood bison navigate trade‐offs in energy gain while foraging we test how their foraging decisions are influenced by environmental factors and constraints within wet meadows (fourth order) where sedge abundance and thus energy is high, but so is predation risk. Specifically, we studied the foraging behavior of a wood bison population, called the Ronald Lake herd, in northeastern Alberta, Canada. The diet of wood bison has been studied extensively, showing selection for sedge species, specifically *C. atherodes*, and a switch to a graminoid‐dominated diet during the forage‐limited winter season (Larter and Gates 1991; Hecker, Edwards, and Nielsen 2021). Our objective was to better understand winter bison foraging ecology, by examining how trade‐offs affect winter foraging decisions. While selecting winter foraging locations, we predicted bison will follow an OFT strategy for resource selection (forage availability model), be affected by energetic trade‐offs and thus follow an EMH strategy (forage accessibility model), try to minimize predation risk when foraging (predation risk model), or manage trade‐offs between these factors (interactive models) (Table 1).

**TABLE 1:** Set foraging hypotheses for wood bison in northeastern Alberta, Canada, framed by ecological theory including a hypothesis title followed by a description of the hypothesis and supporting literature.

Ecological theory	Hypothesis	Prediction	Sources: author(s) (year)^Species^ ^a^
	Null	No pattern in forage site selection.	
Optimal Foraging Theory	Forage availability	Increases in forage availability will increase selection.	Larter and Gates (1991)^1^ Courant and Fortin (2012a)^1^ Courant and Fortin (2012b)^1^
Energy Maximization Hypothesis	Forage accessibility	Decreases in forage accessibility will decrease selection.	Bruggeman et al. (2008)^1^ Doerr, Degayner, and Ith (2005)^4^ Romtveit et al. (2021)^8^
Predation	Predation minimization	Increases in predation risk will decrease selection.	Nelson and Mech (1986)^4/5^
Trade‐off: Predation vs. optimal forage	Forage availability **×** predation minimization	Forage site selection will balance trade‐offs between availability of food and minimizing predation risk.	Hebblewhite and Merrill (2009)^2/5^ Pierce, Bowyer, and Bleich (2004)^4/6^
Trade‐off: energy maximization vs. predation	Forage accessibility **×** predation minimization	Forage site selection will balance trade‐offs between accessibility of food and minimizing predation risk.	Bergman et al. (2006)^1&2/5^
Trade‐off: optimal forage vs. energy maximization	Forage availability **×** forage accessibility	Forage site selection will balance trade‐offs between availability of food and accessibility of food.	Gilbert et al. (2017)^4^ Fortin et al. (2003)^1^ Schaefer and Messier (1995b)^3^ Massé and Côté (2012)^4^ Robinson and Merrill (2012)^2^ Poole and Mowat (2005)^2&4^ Johnson, Parker, and Heard (2000)^7^
Trade‐off: energy maximization vs. predation vs. optimal forage	Complete interaction	Forage site selection will balance trade‐offs between accessibility of food, availability of food, and minimizing predation risk.	Beier and McCullough (1990)^4^

^a^ Superscript species key: 1‐Bison, 2‐Elk, 3‐Muskoxen, 4‐Deer, 5‐Wolf, 6‐Cougar, 7‐Woodland Caribou, 8‐Mountain Reindeer.

## 2 Methods

### 2.1 Study System

**FIGURE 1:**
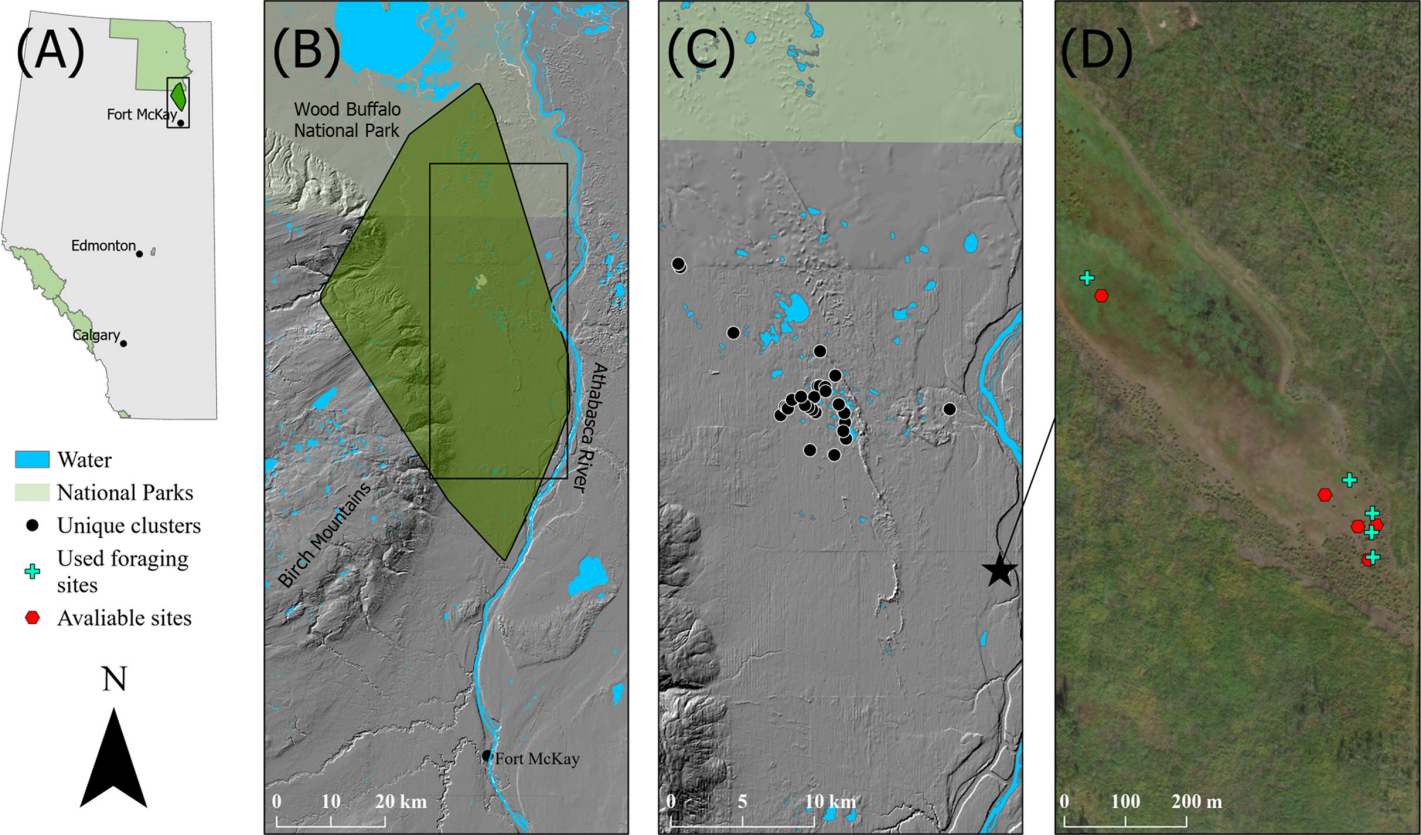
Location of the Ronald Lake wood bison annual home range (100% minimum convex polygon) northeastern Alberta, Canada, (A) between the Athabasca River and the Birch Mountains (B; DeMars, Nielsen, and Edwards 2020). Location of sampled cluster sites in wetlands within 25 km of Ronald Lake (C) and an example the matched‐paired designing within an individual cluster site (D) with forage sites (crosses) and available random pairs (hexagons) displayed.

We studied winter habitat selection in a small population of wood bison (~272 individuals) (S. Young, email message, September 13, 2021) located near Ronald Lake, Alberta, Canada. The population is approximately 90‐km north of Fort McKay, with an annual range of 2200‐km^2^. Their range is bound by the Athabasca River in the east, the Birch Mountains in the west, the southeastern part of Wood Buffalo National Park in the north, and the northern extent of oil sands development in the south (Figure 1B). Located in the River Lowlands of the boreal forest ecoregion, the range is dominated by upland forest (37% upland deciduous, 23% upland conifer), with a network of wetlands (38% peatlands/swamps, 4% graminoid rich meadows) and small lakes (4% open water) throughout (Ducks Unlimited Canada 2016; Hecker, Edwards, and Nielsen 2021). Upland habitats are dominated by quaking aspen (*Populus tremuloides*), jack pine (*Pinus banksiana*), and white spruce (*Picea glauca*) (DeMars, Nielsen, and Edwards 2020). The graminoid‐rich meadows are dominated by three *Carex* species (*C. aquatilis*, *C. atherodes*, and *C. utriculata*). The average annual snowfall of from 1990 to 2020 in the area is 110.6 cm, according to the closest Government of Canada weather station, 150 km south of Ronald Lake, in Fort McMurray Alberta, Canada (Government of Canada 2024). The area also has a network of legacy seismic lines used by the energy industry to explore oil and gas and a more concentrated density of recent seismic lines in the southern parts of the range (DeMars, Nielsen, and Edwards 2020).

**FIGURE 2:**
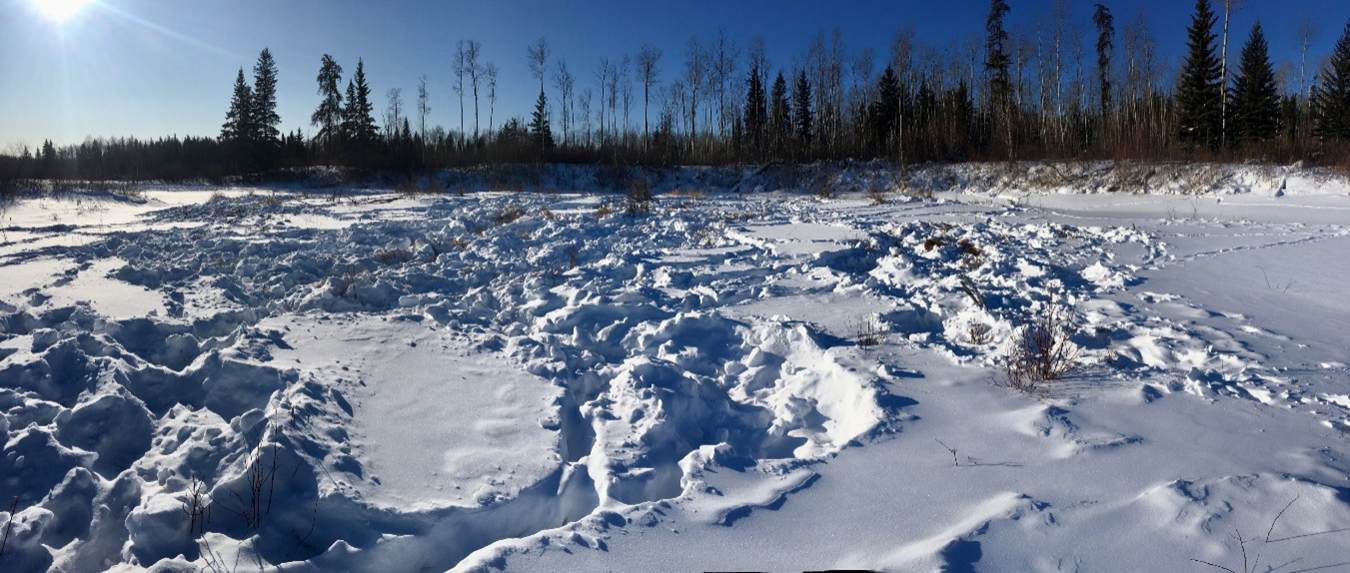
Cluster of winter bison foraging locations, known as craters, in the Ronald Lake region of northeastern, Alberta, Canada.

Between 2013 and 2021, 70 of the herd's females were fitted with Vectronics Vertex Plus Global Positioning System (GPS) radio collars (VECTRONIC Aerospace, Carl‐Scheele‐Str. 12, 12489 Berlin, Germany) by the Government of Alberta programmed to acquire location data every 90 min. Using recorded GPS locations, we quantified bison foraging behavior in recently visited (within 7 days) wetlands during the winters (January–March) of 2020 and 2021. Any wetland within 25 km of Ronald Lake that exhibited bison activity was selected for study (Figure 1). Field locations were based on identified clusters of bison from GPS collar data, with recent (within 7 days) areas of bison activity sampled. GPS clusters were verified as foraging locations by the presence of snow craters, tracks, and vegetation disturbance (Figure 2). All individual craters were sampled separately and paired with an available random site within the same wetland that showed no foraging sign on the same date, based on a matched‐pair study design. Available sites were all 0.25 m^2^ and were selected using a random bearing and distance from the accompanying used site, however, available sites were required to be within the same wetland and could not show signs of use. If the random bearing and direction led to a site that did not satisfy these conditions a new bearing and direction were selected.

### 2.2 Optimal Foraging Theory

#### 2.2.1 Forage Availability

We recorded vegetation characteristics at each location to test the influence of different food on forage site selection. *Carex* species, specifically *C. aquatilis*, *C. atherodes*, and *C. utriculata*, are abundant in the Ronald Lake region and are known to be selected by bison (Larter and Gates 1991; Courant and Fortin 2012a). These key foods were identified at the species level using morphological structures and seed heads present beneath the snow, while grasses (*Poa* spp.) and cattail (*Typha* spp.) were identified to the genus level, and all woody plants were described as browse. In a few cases when *Carex* spp. were not able to be identified, the site was revisited the following summer to verify the selected species. After identifying vegetation present in an individual crater, we visually estimated the species composition which was represented by the percent ground cover of each plant per crater. Foraging intensity was also measured as a numeric ordinal class from zero to four, with zero representing no foraging and four representing complete foraging to the ground level (Holecheck and Galt 2000). Models testing forage availability included the percent ground cover of the three *Carex* species, grasses, cattails, and browse, only excluding records with intensity scores of 0, as they were not actively selected.

### 2.3 Energy Maximization

#### 2.3.1 Forage Accessibility

Snow in northern climates has been shown to shape ungulate movement and foraging behavior (Bruggeman et al. 2008; Sheppard et al. 2021). For bison, snow characteristics alter the amount of energy required to reach forage species, with deeper, harder snow requiring increased energy to penetrate and uncover food (Bruggeman et al. 2008). To capture a representation of the snow conditions present during a foraging event, we measured depth of crust, total depth, snow water equivalent (SWE) of crust, total SWE, and crust hardness, by digging one to three snow pits, dependent on crater size, at the crater edge and repeated the measurements at each associated available pair (Fortin 2005). Within each pit, snow crust and total depth were measured to the nearest 0.5 cm using a meter stick; then, using a Snowmetrics snowboard sampler, we measured the SWE of the crust and total snow column in each pit. Later these SWE measurements were converted to snow density (Snowmetrics 2021). We then recorded the hardness of the snow crust based on the hand hardness test, which uses objects of decreasing areas (1 = fist, 2 = four fingers, 3 = one finger, 4 = pencil, 5 = knife) to press into the snow and scores hardness based on which object breaks the crust (Höller and Fromm 2010). In the region, snow generally accumulates throughout November—March before melting in early April. Bison selected sites ranged in snow depth from 13 to 67 cm, while random paired sites ranged from 17 to 97 cm. After testing for correlation of snow variables total depth was selected to represent the snow condition as it best explained selection when combined with forage availability and predation risk.

### 2.4 Minimizing the Predation Risk

#### 2.4.1 Predation Risk

Ungulates, including bison, alter foraging behavior to minimize the risk of predation (Nelson and Mech 1986; Harvey and Fortin 2013). For example, bison tend to avoid large open areas or stay closer to the forest edge to minimize the predation risk from gray wolves (Simon, Cherry, and Fortin 2019). As such, distance to forest cover or wetland edge can be used as a proxy for predation risk, with increased distance from the forest edge increasing the potential for predation (Hernández and Laundré 2005; Bergman et al. 2006; Creel, Schuette, and Christianson 2014). Using aerial imagery, we calculated the distance to cover in ArcGIS Pro, first digitizing sampled wetlands at a 1:2500 scale (Esri 2022). We then used the Near Tool to calculate the distance from each recorded site (used and available) to the edge of the digitized polygon based on GPS points taken from the center of each crater when visited.

### 2.5 Individual Versus Interactive Effects

We tested eight a priori hypotheses predicting patterns of selection, including a null model where forage site selection was unaffected by any environmental factors, a selection of models that test individual foraging influence factors, and a selection of models that combine the individual factors using interactive affects (Table 1). Using the *mclogit* package in R, in accordance with our matched paired study design and field methods, we fit case–control conditional logistic regression models to each hypothesis, with each selected foraging site (case) being compared to the paired available site (control) (Elff 2022; Therneau 2023; R Core Team 2022). Conditional logistic regression allows for the comparison of sites while controlling for seasonal changes in conditions and potential re‐use of selected wetlands by comparing temporally related observations. Conditional logistic regression also minimizes autocorrelation as each pair is compared individually within designated strata, thus minimizing the influence of spatial patterns of sampled sites (Compton, Rhymer, and McCollough 2002). We evaluated support for each hypothesis based on the principle of parsimony using the change in Akaike's information criteria (ΔAIC) from the model with the lowest overall AIC score and then also examining each models Akaike weight (*w*_*i*_) (Akaike 1974; Wagenmakers and Farrell 2004). Effect size is expressed as odds ratios (e^β) for each model term, which can be interpreted as X percent increase in predicted selection based on a single unit increase in a predictor and is calculated by exponentiating the β‐coefficient of a logistic regression. Standard errors (SE) are also presented for each variable or interaction of variables within the most supported model.

## 3 Results

### 3.1 Trade‐Offs in Foraging Site Selection

In total, we visited 190 paired sites of used and available foraging locations across 20 unique wetlands during January—March 2020 and 2021. This included 36 unique clusters ranging from 2 to 25 individual foraging events. Models comparing bison selection of foraging patch during winter supported trade‐offs between selecting sites with optimal forage available and maximizing energy by avoiding deep snow (Table 2). In fact, the three most supported models all highlight trade‐offs between forage availability and other factors, with the most parsimonious model supporting the interactive effect of forage accessibility (snow condition) and forage availability, with an Akaike weight of 0.56 (Table 2). The model testing only forage availability, closer to an OFT strategy, ranked fourth with an Akaike weight of 0.05, following models supporting the interactions between forage availability and minimizing predation (*w*_*i*_ = 0.29), and the interactions between forage availability, forage accessibility, and predation risk (*w*_*i*_ = 0.10), (Table 2). Despite similar support for both top two models, the forage accessibility and forage availability model was the most supported based on Akaike weight with a *w*_*i*_ = 0.56, compared to the second‐ranked forage availability and predation minimization model with a *w*_*i*_ = 0.29.

**TABLE 2:** Comparison of candidate models explaining the forage site selection of wood bison in northeastern Alberta, Canada. Model selection results listing model complexity (*K*), Akaike's information criteria (AIC), change in AIC (ΔAIC) and overall supports (*w*_*i*_) of the models. Models are ranked in order of most to least supported.

Model	Model structure^a^	*K*	AIC	ΔAIC	*w*_*i*_
Forage availability × forage accessibility	Selection ~ forage species cover (%) **×** total snow depth (cm)	15	135.95	0.00	0.56
Forage availability × predation minimization	Selection ~ forage species cover (%) **×** distance to cover (m)	13	137.23	1.28	0.29
Forage availability × forage accessibility × predation minimization	Selection ~ forage species cover (%) **×** total snow depth (cm) **×** distance to cover (m)	25	139.37	3.42	0.10
Forage availability	Selection ~ forage species cover (%)	8	140.64	4.68	0.05
Forage accessibility × predation minimization	Selection ~ total snow depth (cm) × distance to cover (m)	5	209.02	73.07	< 0.01
Forage accessibility	Selection ~ total snow depth (cm)	3	214.49	78.54	< 0.01
Predation minimization	Selection ~ distance to cover (m)	3	251.27	115.32	< 0.01
Null		2	263.40	127.45	< 0.01

^a^ Forage species cover (%) includes percent ground cover of vegetation group, including *Carex atherodes*, *Carex aquatilis*, *Carex utriculata*, *Poa* spp., *Typha* spp., and browse spp. as individual parameters in the model.

### 3.2 Environmental Factors Affecting Forage Selection

Models comparing environmental factors on wood bison winter foraging site selection strongly supported the interactive effect of forage accessibility, measured through snow condition, and forage availability (ΔAIC of 127.45 with null model). When considering only individual variables, forage species availability was the most supported factor influencing bison forage site selection with a ΔAIC of 4.68, while forage accessibility had a ΔAIC of 78.54. These models show the importance of competing factors influencing behavior, as individual snow and forage models lead to different conclusions than when considered interactively.

### 3.3 Forage Availability and Accessibility

Model parameters for the most supported model confirm the expected selection of areas with high percent cover of sedge species (*C. atherodes* odds ratio [e^β] = 1.21, *p* = < 0.01; *C. aquatilis* odds ratio [e^β] =1.17, *p* = 0.01; *C. utriculata* odds ratio [e^β] = 1.17, *p* = 0.01). Total snow depth alone did not significantly influence site selection. There was an inverse relationship between forage site selection and food cover at higher snow depths (Table 3).

**TABLE 3:** Model parameters predicting selection of foraging sites by wood bison in northeastern Alberta, Canada, as a function of snow conditions and food percent cover, including betas (β), standard error (SE), *Z*‐score (*Z*) and associated *p*‐value (*p*) (significant variables bolded *p* < 0.05), and odds ratio (e^β).

Model variable	*Β*	SE	*Z*	*p*	e^β
*C. atherodes* cover (%)	**0.19**	**0.07**	**2.873**	**< 0.01**	**1.21**
*C. aquatilis* cover (%)	**0.16**	**0.07**	**2.486**	**0.01**	**1.17**
*C. utriculata* cover (%)	**0.16**	**0.06**	**2.501**	**0.01**	**1.17**
*Poa* spp. cover (%)	**0.15**	**0.06**	**2.317**	**0.02**	**1.16**
Browse spp. cover (%)	0.09	0.21	0.436	0.66	1.10
*Typha* spp. cover (%)	0.15	0.13	1.181	0.24	1.17
Total snow depth	0.18	0.11	1.614	0.11	1.19
*C. atherodes* × snow depth	**< −0.01**	**< 0.01**	**−2.197**	**0.03**	**0.99**
*C. aquatilis* × snow depth	**< −0.01**	**< 0.01**	**−2.037**	**0.04**	**0.99**
*C. utriculata* × snow depth	**< −0.01**	**< 0.01**	**−1.973**	**0.05**	**0.99**
*Poa* spp. × snow depth	**< −0.01**	**< 0.01**	**−2.432**	**0.02**	**0.99**
Browse spp. × snow depth	< −0.01	< 0.01	−0.825	0.41	0.99
*Typha* species × snow depth	< −0.01	< 0.01	−1.342	0.18	0.99

**FIGURE 3:**
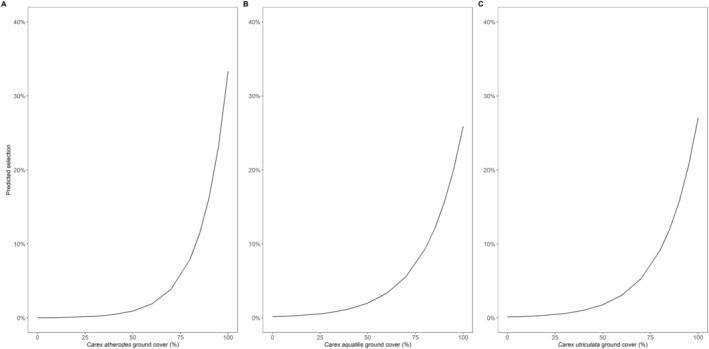
Marginal effect curves for *Carex atherodes* (A), *C. aquatilis* (B), and *C. utriculata* (C) from the most supported model predicting bison selection of foraging locations in northeastern Alberta, Canada.

**FIGURE 4:**
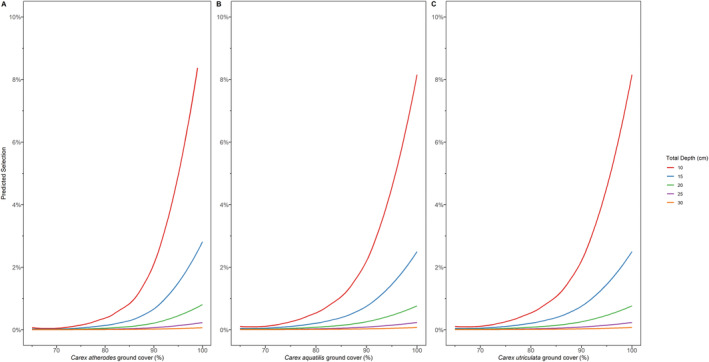
Marginal effect curves for the interactions between total snow depth and *Carex atherodes* (A), *C. aquatilis* (B), and *C. utriculata* (C) from the most supported model predicting bison selection of foraging locations in northeastern Alberta, Canada.

The marginal effects of the main variables in the most supported model show sharp increases in predicted selection for each of the sedge species at around 50% ground cover (Figure 3). Conversely, the likelihood of selection decreases rapidly at snow depths between 10 and 30 cm (Figure 4). While all sedge species show similar selection at 50% ground cover (*C. atherodes* selection = 1% *C. aquatilis* selection = 2%, *C. utriculata* selection = 2%), *C. atherodes* shows a higher predicted selection at 100% cover (33% selection) than *C. aquatilis* and *C. utriculata* (26% selection, 27% selection, respectively, Figure 3).

## 4 Discussion

In this paper we support the idea that winter foraging behavior of wood bison is influenced not only by food availability, but also by the environmental conditions present when foraging. Our results provide further support for the energy maximization hypothesis, which posits organism‐manage trade‐offs between energy intake and energy expenditure to optimize energetic level. Indeed, forage availability and quality explain many of the foraging behaviors of ungulates (Fortin 2005; Courant and Fortin 2012a). For example, white‐tailed deer (*Odocoileus virginianus*) select habitat based on the highest likelihood of food availability (Massé and Côté 2012). Our results support bison selection of *Carex* spp. during winter foraging (Larter and Gates 1991; Courant and Fortin 2012a) and shows a clear increase in predicted selection of foraging sites as the cover of *Carex* spp. increases, particularly above 50% (Figure 3). Each *Carex* spp. had a positive effect on selection, with a 1% increase in *C. atherodes* ground cover leading to an increase in selection by a factor of 1.21 (odds ratio [e^β] = 1.21, Table 3). The same increase in *C. utriculata* or *C. aquatilis* ground cover led to an increase in selection by a factor or 1.17 (odds ratio [e^β] = 1.17; Table 3). These trends highlight the importance of quality forage, as *Carex* spp. are a source of high digestible energy in the Ronald Lake range during the otherwise limited winter season (Fortin, Fryxell, and Pilote 2002). It follows that bison select foraging sites based on the type of food present (Courant and Fortin 2012a). However, when interactions with snow depth are considered, we observe more of an energy maximizing response.

Winter conditions constrain bison from following a traditional OFT approach to foraging behavior, and instead they follow an energy maximizing strategy. Importantly, the combination of both food availability and snow conditions best explains forage site selection, showing that bison are influenced by multiple factors when selecting foraging sites. If food availability was the key explanatory variable, we would expect *C. atherodes* availability to be the most strongly associated with winter forage site selection, regardless of environmental conditions (Larter and Gates 1991). In contrast, our results showed that bison limit their energy expenditure while foraging by avoiding energy‐intense searching behavior, i.e., avoiding areas with deep snow. For example, the usual increase in selection by a factor of 1.21 with 1% increase in *C. atherodes* ground cover is neutralized when snow depths are also increasing (e^β = 0.99; Table 3). Minimizing the cost of deep snow is displayed in many other ungulates that experience winter conditions. Both wood bison and elk display decreased movement rates to conserve energy in deep snow (Sweeney and Sweeney 1984; Sheppard et al. 2021). When foraging, mountain reindeer (*Rangifer tarandus*) select snow‐free habitats to conserve energy rather than cratering; similarly, muskoxen increase residency times and foraging effort at increased snow depths (Schaefer and Messier 1995a; Romtveit et al. 2021). In our case, when snow condition is considered with vegetation cover, a similar relationship is seen for all *Carex* spp. (Table 3). Bison are less likely to select areas to forage that have deeper snow, regardless of the *Carex* spp. availability, as shown by the rapid decrease in predicted selection around as snow depth increases (Figure 4). Similar to Sheppard et al. (2021), who found bison movement rate decreased substantially when snow depth was more than 40 cm, we suggest that bison altered their behavior to avoid expending energy in areas of deep snow. There is likely little nutritional difference between *Carex* spp. during winter, and as such, it is not energetically efficient to dig in deeper snow to access otherwise selected species, like *C. atherodes*. Alternatively, the digestive limitations of bison combined with variability in snow conditions could allow bison to access all required food in shallow snow within each selected wetland.

In contrast to our predictions, we found that our estimation of predation risk was not a significant factor influencing the foraging decisions of wood bison. Many ungulate species, including bison, alter their behavior to minimize predation risk (Laundré et al. 2001). Some common risk minimizing behaviors include selecting low risk foraging locations at the expense of forage quality or reducing patch residency times when in high‐risk locations (Hebblewhite and Merrill 2009; Courant and Fortin 2012b). These behaviors may not be detectable when comparing foraging decisions within singular wetlands, as residency time and comparative forage quality to other possible wetlands were not measured explicitly. Perhaps, as Hernández and Laundré (2005) found, bison behavior is not significantly altered by wolf presence as the non‐primary target of wolf predation. Simon, Cherry, and Fortin (2019) showed that plains bison forage site quality was negatively affected by high predation risk during the spring and summer, but winter saw bison select high‐quality forage across predation risk differentials. This paper highlights how in higher‐risk seasons, when winter is energetically taxing, bison were more likely to risk predation to obtain high‐quality forage. Dewart (2023), highlighted bison predation on the Ronald Lake herd to be rare, with all occurrences of predation happening after snow depth was greater than 30 cm, and highlighted prey switching to less dangerous prey when available (Tallian et al. 2017). The small pack size of wolves in the Ronald Lake area could also lead to less predation on bison, leading to less predation pressure overall (MacNulty et al. 2014; Dewart 2023). Alternatively, the behaviors that did explain bison foraging at a fine scale, including avoidance of deep snow, could minimize predation risk. Decreasing energy‐intensive foraging behavior allows wood bison to increase vigilance, and spending less time digging through deep snow could also limit patch residency time and by extension limit predation risk (Harvey and Fortin 2013). Further observational study of the risk‐minimizing behaviors of the Ronald Lake bison herd could further explain how they navigate the ‘landscape of fear’ (Laundré, Hernández, and Altendorf 2001).

Many studies tend to focus on forage quantity and quality as key factors influencing animal foraging behavior. We demonstrate the importance of considering other energy constraints animals encounter when searching for or acquiring food. We also highlight how the interactions of these constraints, in this case, snow conditions, with forage availability is more informative than focusing solely on available energy from food. To fully understand animal foraging ecology, we suggest examining all factors that affect individual foraging decisions. Our study demonstrates that while OFT is a staple of ecological theory and shows merit in many instances, it is imperative to consider competing factors when assessing foraging behavior.

## Author Contributions

**Garrett J. Rawleigh:** conceptualization (lead), data curation (lead), formal analysis (lead), funding acquisition (supporting), investigation (lead), methodology (lead), validation (lead), visualization (lead), writing – original draft (lead), writing – review and editing (equal). **Mark A. Edwards:** conceptualization (supporting), formal analysis (supporting), funding acquisition (lead), investigation (supporting), methodology (supporting), project administration (lead), resources (lead), supervision (lead), validation (equal), visualization (supporting), writing – review and editing (equal). **Darren Epperson:** conceptualization (supporting), investigation (supporting), methodology (supporting), project administration (supporting). **Scott E. Nielsen:** conceptualization (equal), formal analysis (supporting), funding acquisition (lead), investigation (supporting), methodology (equal), project administration (lead), resources (lead), software (lead), supervision (lead), validation (equal), visualization (supporting), writing – review and editing (equal).

## Conflicts of Interest

The authors declare no conflicts of interest.

## Data Availability

Data supporting the findings of this study are available on Dyrad at: https://datadryad.org/stash/share/mSg3QygZT33uj2jBDcRHQ6NX5a7YHCoakSOO09aCW_A.
